# Repeat emergency department visits by nursing home residents: a cohort study using health administrative data

**DOI:** 10.1186/s12877-018-0854-8

**Published:** 2018-07-05

**Authors:** Andrea Gruneir, Candemir Cigsar, Xuesong Wang, Alice Newman, Susan E. Bronskill, Geoff M. Anderson, Paula A. Rochon

**Affiliations:** 1 Department of Family Medicine, University of Alberta, 6-10 University Terrace, Edmonton, AB T6G 2T4 Canada; 2 Mathematics and Statistics, Memorial University of Newfoundland, HH-3046, St. John’s, NL A1C 5S7 Canada; 3 Institute for Clinical Evaluative Sciences, G1 06, 2075 Bayview Ave, Toronto, ON M4N 3M5 Canada; 4 Institute of Health Policy, Management and Evaluation, University of Toronto, 155 College St. Suite 425, Toronto, ON M5T 3M6 Canada; 5 Women’s College Research Institute, Women’s College Hospital, 76 Grenville St, Toronto, ON M5S 1B2 Canada

**Keywords:** Repeated events, Recurrent events, Long-term care, Emergency, Transfers

## Abstract

**Background:**

Nursing home (NH) residents are frequent users of emergency departments (ED) and while prior research suggests that repeat visits are common, there is little data describing this phenomenon. Our objectives were to describe repeat ED visits over one year, identify risk factors for repeat use, and characterize “frequent” ED visitors.

**Methods:**

Using provincial administrative data from Ontario, Canada, we identified all NH residents 65 years or older who visited an ED at least once between January 1 and March 31, 2010 and then followed them for one year to capture all additional ED visits. Frequent ED visitors were defined as those who had 3 or more repeat ED visits. We used logistic regression to estimate risk factors for any repeat ED visit and for being a frequent visitor and Andersen-Gill regression to estimate risk factors for the rate of repeat ED visits.

**Results:**

In a cohort of 25,653 residents (mean age 84.5 (SD = 7.5) years, 68.2% female), 48.8% had at least one repeat ED visit. Residents who experienced a repeat ED visit were generally similar to others but they tended to be slightly younger, have a higher proportion male, and a higher proportion with minimal cognitive or physical impairment. Risk factors for a repeat ED visit included: being male (adjusted odds ratio 1.27, (95% confidence interval 1.19–1.36)), diagnoses such as diabetes (AOR 1.28 (1.19–1.37)) and congestive heart failure (1.26 (1.16–1.37)), while severe cognitive impairment (AOR 0.92 (0.84–0.99)) and 5 or more chronic conditions (AOR 0.82 (0.71–0.95)) appeared protective. Eleven percent of residents were identified as frequent ED visitors, and they were more often younger then 75 years, male, and less likely to have Alzheimer’s disease or other dementias than non-frequent visitors.

**Conclusions:**

Repeat ED visits were common among NH residents but a relatively small group accounted for the largest number of visits. Although there were few clear defining characteristics, our findings suggest that medically complex residents and younger residents without cognitive impairments are at risk for such outcomes.

**Electronic supplementary material:**

The online version of this article (10.1186/s12877-018-0854-8) contains supplementary material, which is available to authorized users.

## Background

Emergency departments (EDs) are important sites of care for nursing home (NH) residents; however, the frequency at which residents are transferred to EDs has been cause for concern and has raised questions about the extent to which such transfers are appropriate or avoidable [1]. Substantial research has sought to quantify the frequency of ED visits from NHs, in particular those that may be amenable to preventive interventions. Despite the volume of research, little attention has been paid to the issue of repeat ED visits, that is individual residents with multiple transfers, even though early studies reported a greater number of visits than residents in their cohorts [2, 3]. More recent studies report similar findings, including a study using National Hospital Ambulatory Care Survey data that reported an average of 1.8 visits per resident per year [4]. Beyond such simple statistics, there is little data describing the frequency of repeat ED visits from NHs or the residents who make multiple visits.

In the general (non-NH) population, a small proportion of individuals (4–8%) accounts for a relatively large share of all ED visits [5, 6]. This group of “frequent ED users” has been shown to be fairly heterogeneous but are more likely to experience poor physical and mental health, and problems with substance abuse than other ED users [7–9]. Frequent ED use does not appear to be associated with insurance coverage or having a regular source of care [10, 11]. Among older adults in the general population, nearly 15% return to the ED within 30 days of an initial visit. For this group, return to the ED is associated with cognitive impairment, multiple chronic conditions, depression, and a history of prior ED use [12].

NH residents are a highly vulnerable group characterized by older age, multiple chronic conditions, and advanced cognitive and physical impairments. A better understanding of outcomes, such as repeated ED transfers, is critical to ensuring improved care for residents. By identifying how frequently repeated ED transfers from NHs occur and which residents are at greatest risk, we can begin to identify when and where interventions to improve resident care may be most successful in reducing preventable ED transfers. In this study, we had three objectives: 1) to describe all repeat ED transfers made by a cohort of LTC residents over one year; 2) to identify resident characteristics associated with repeat ED transfer; and 3) to characterize a group of “frequent ED users” defined as residents who experienced 4 or more ED transfers within the year.

## Methods

### Setting

This study was conducted in Ontario, Canada’s largest province. Ontario has a population of approximately 13 million people, with nearly 75,000 living in a NH at any given point in time. NHs are residential settings that provide access to 24-h care for adults unable to live safely in community settings. Most NH beds are used for long-term care purposes with limited use for post-acute or rehabilitative care. In Ontario, the vast majority of individuals have publicly funded health insurance, which covers physician visits, acute care, and NH care.

### Study design and data

This is a retrospective cohort study using multiple health administrative databases, including: the Continuing Care Reporting System (CCRS), the National Ambulatory Care Reporting System (NACRS), the Discharge Abstract Database (DAD), and the Registered Persons Database (RPDB). The CCRS is a repository of Resident Assessment Instrument – Minimum Data Set, version 2.0 (RAI-MDS 2.0) clinical assessments that are mandated for completion on all NH residents in Ontario. The RAI-MDS 2.0 includes over 300 data items on diagnoses, cognitive and physical functioning, and behaviour. NACRS is mandated for completion on all visits to EDs within the province and includes various time and date stamps, and diagnoses. The DAD consists of standardized chart abstractions of all acute care hospital stays. The RPDB contains basic demographic data, including dates of birth and death (if applicable), for all provincial residents with valid health insurance. These datasets were linked using encoded identifiers and analyzed at the Institute for Clinical Evaluative Sciences (ICES).

### Ethics

ICES is a prescribed entity under section 45 of Ontario’s Personal Health Information Protection Act. Section 45 authorizes ICES to collect personal health information, without consent, for the purpose of analysis or compiling statistical information with respect to the management of, evaluation or monitoring of, the allocation of resources to or planning for all or part of the health system. Projects conducted under section 45, by definition, do not require review by a Research Ethics Board. This project was conducted under section 45 and approved by ICES’ Privacy and Compliance Office. The study received approval from the Research Ethics Board at Women’s College Hospital, Toronto, Canada.

### Cohort identification and follow-up

**Fig. 1:**
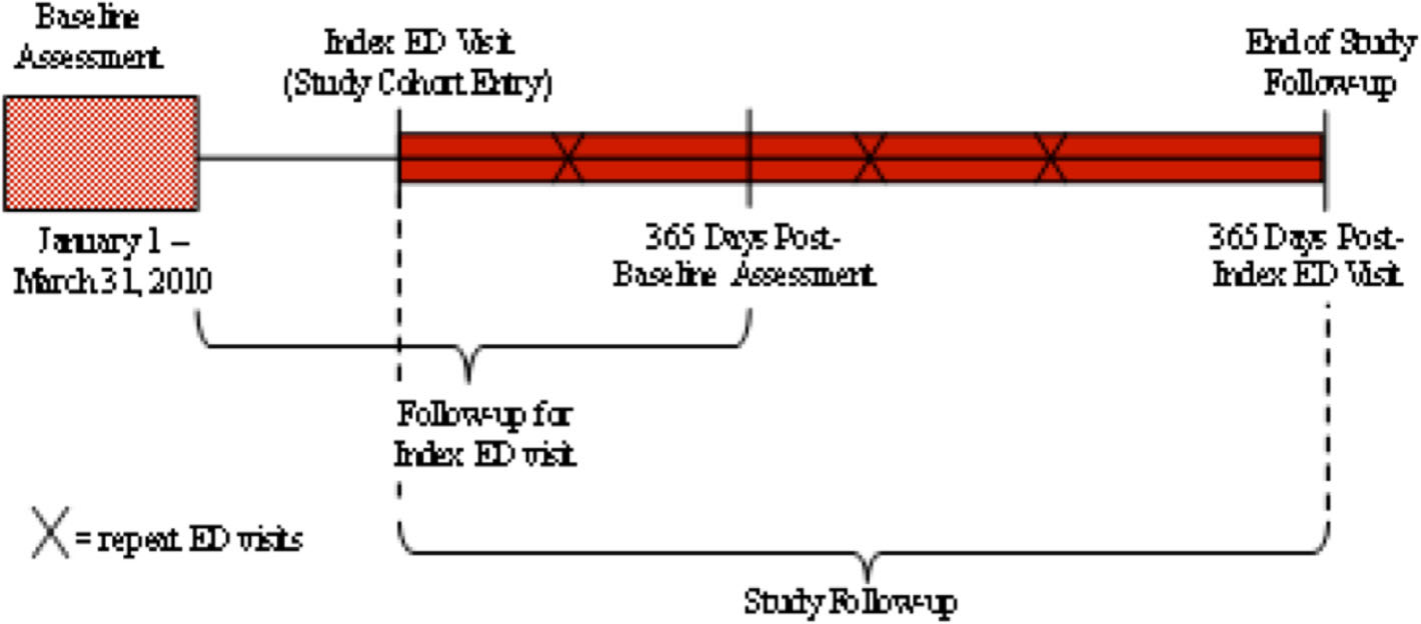
Cohort development and timeline

We identified all individuals who resided in a NH in Ontario between January 1 and March 31, 2010, using the first RAI-MDS assessment (if more than one) from this period as baseline. We excluded residents younger than age 65 years (*n* = 4868) since they have different health needs which may influence their use of acute care services. We further excluded individuals who were not Ontario residents (139), had invalid unique identifiers (26), or resided in a NH with fewer than 25 beds (323). This left a total of 71,766 residents. From baseline, residents were followed to their first ED visit (the “index” visit) or the end of 365 days, whichever came first. We identified 25,897 residents in 604 NHs who had an index ED visit within one year of baseline. Of this group, 246 (0.9%) died during the index ED visit and were excluded from further analyses. The final cohort consisted of 25,653 individual residents who were discharged alive from an index ED visit. This cohort was followed from the date of the index ED visit until the first of: discharge from the LTC home, death, or 365 days from the index ED visit. All subsequent ED transfers and acute care hospital admissions within the follow-up year were counted and treated as risk-free periods. See Fig. 1.

### Resident characteristics

All variables used to describe residents were obtained from the baseline RAI-MDS 2.0 assessment. This included age, sex, length of stay in the NH at baseline, clinical conditions, cognition, physical functioning, and behaviour. Cognition was measured using the embedded Cognitive Performance Scale [13]. Physical functioning was assessed on the Activities of Daily Living (ADL) Short-Form Scale [14]. We also included the Changes in Health and End-Stage Signs and Symptoms (CHESS) Scale as a measure of clinical instability [15]. To estimate multimorbidity, we summed clinical conditions. As a sensitivity analysis, we assessed for changes in resident characteristics from baseline to subsequent RAI-MDS 2.0 assessments but found little change.

### Emergency department visits

We identified all unscheduled ED visits that occurred between the index ED visit and the end of the follow-up period. ED visits were described according to diagnosis, type, timing, and discharge disposition. All visits were classified into one of five mutually exclusive categories based on diagnosis to obtain visit type, as defined in our earlier work [1, 16]. These were: potentially preventable (visits which may have been avoided had a pre-existing condition been better managed earlier, comparable to ambulatory care sensitive conditions), fall-related injury, non-fall injury, low acuity (visits that were triaged as non-urgent and ended with the resident returning to the LTC home), and other (visits that did not fit in any of the other categories). The timing of each visit was defined according to the day of week and time of day (weekday, weeknight, weekend-day, and weekend-night) recorded at triage. Discharge disposition captured the resident’s discharge location and included admission to hospital, death within the ED, and return to the NH.

All visits, except for the index ED visit, were also characterized by the number of days since discharge from the preceding ED visit and categorized as: immediate (within 72 h of discharge from the preceding ED visit), short-term (within 3–10 days), long-term (within 11–90 days), and distant (91 or more days), as adapted from Weiss and colleagues [5]. We also looked to see if visits were concordant on type with the ED visit immediately preceding it.

### Frequent ED visitors

We identified a subset of residents who could be described as “frequent ED visitors”. To be consistent with the research on non-NH populations, these were defined as residents who had 4 visits within one year (in this case, those with 3 repeat ED visits) [6].

### Analyses

We characterized the cohort according to baseline demographic, clinical, and functional characteristics, as well as the index ED visit. We counted all subsequent (repeat) ED visits and categorized as one of: second, third, fourth, or fifth or later visits; we used descriptive statistics to fully characterize all repeat ED visits.

We followed a series of steps to identify risk factors for repeat ED visits. First, we performed bivariate statistical analyses to compare residents who did and did not experience any repeat ED visits on baseline characteristics and index ED visit characteristics. We then used logistic regression to identify risk factors for making at least one repeat ED visit. We modeled a multinomial outcome: any repeat ED visit, death without a repeat ED visit, and no repeat ED visit or death (the reference category). Each variable that significantly differed on bivariate comparisons was tested in the multinomial logistic regression. Any variable that did not reach a significance level of *p* < 0.05 was removed from the model. Next, we used an Andersen-Gill (A-G) model to estimate the effect of identified risk factors on the rate of repeat ED visits [17]. The A-G model is a generalization of the Cox proportional hazards model extended for recurrent outcome events and is recommended based on its efficiency and power [18, 19]. In this model, the dependent (outcome) variable is the rate of repeat ED visits measured as the time between visits counted over the follow-up period. The model provides maximum likelihood estimates of a relative rate (RR) and 95% confidence interval (95% CI) for each independent variable (in this case, the risk factors selected from the logistic regression models). The RR quantifies the change in the repeat ED visit rate relative to a baseline rate. Other benefits of this model include its ability to account for risk-free time (for example, when a resident is in the ED), clustering in NHs, and competing risks (specifically death).

Frequent visitors, those who experienced 3 or more repeat ED visits, were compared to all other residents in the cohort on baseline characteristics. Multinomial logistic regression was used to identify risk factors for being a frequent ED visitor. The model outcomes were: 3 or more repeat ED visits, death, or fewer than 3 repeat ED visits (reference). Risk factors were identified in a step-wise fashion by first testing variables in bivariate analyses, then added in categories to a single model, then a full model, and finally a reduced model based on associations identified as significant at *p* < 0.05. Repeat ED visits among frequent visitors were described in a manner similar to that for the full cohort.

All analyses were conducted in SAS version 9.4.3 except for the A-G model which was conducted in Rstudio version 0.98.1091.

## Results

### The cohort

Among our cohort of 25,653 residents, 12,505 (48.8%) made a total of 24,389 repeat ED visits during the follow-up year. Of residents with repeat ED visits, 6604 (52.8%) made one, 3042 (24.3%) made two, 1425 (11.4%) made three, and the remainder made four or more. A total of 10,310 residents died within the one-year follow-up.

The cohort’s mean (SD) age was 84.5 (7.5) years, 68.2% were female, and 67.9% had been in the NH for one year or longer (Table 1). Fifty-four percent experienced moderate or severe cognitive impairment, and 85.0% experienced moderate to full ADL dependence. The most common reported clinical conditions were non-Alzheimer’s dementias (42.4%), depression (28.3%), diabetes (28.2%), and stroke (22.2%). The majority had more than one chronic condition.

**Table 1:** Descriptive characteristics of study cohort at baseline

	Full cohort *N* = 25,653	No repeat ED visit during follow-up *N* = 13,148	At Least 1 repeat ED visit during follow-up *N* = 12,505
Age, n (%)			
65–74	2782 (10.8%)	1233 (9.4%)	1549 (12.4%)
75–84	9075 (35.4%)	4408 (33.5%)	4667 (37.3%)
85–94	11,784 (45.9%)	6284 (47.8%)	5500 (44.0%)
95+	2012 (7.8%)	1223 (9.3%)	789 (6.3%)
Women, n (%)	17,504 (68.2%)	9238 (70.3%)	8266 (66.1%)
Length of Stay in Nursing Home, n (%)			
< 30 days	775 (3.0%)	318 (2.4%)	457 (3.7%)
30–89 days	1250 (4.9%)	565 (4.3%)	685 (5.5%)
90–364 days	6214 (24.2%)	2904 (22.1%)	3310 (26.5%)
365 or more days	17,414 (67.9%)	9361 (71.2%)	8053 (64.4%)
Cognitive Impairment, n (%)			
Minimal	11,851 (46.2%)	5602 (42.6%)	6249 (50.0%)
Moderate	9013 (35.1%)	4784 (36.4%)	4229 (33.8%)
Severe	4789 (18.7%)	2762 (21.0%)	2027 (16.2%)
Activities of Daily Living Impairment, n (%)			
Minimal	3842 (15.0%)	1817 (13.8%)	2025 (16.2%)
Moderate	10,785 (42.0%)	5217 (39.7%)	5568 (44.5%)
Dependent	11,026 (43.0%)	6114 (46.5%)	4912 (39.3%)
Problem Behaviours, n (%)			
Inappropriate	4337 (16.9%)	2344 (17.8%)	1993 (15.9%)
Verbally abusive	4954 (19.3%)	2590 (19.7%)	2364 (18.9%)
Physically abusive	2859 (11.1%)	1568 (11.9%)	1291 (10.3%)
Wanderer	4641 (18.1%)	2396 (18.2%)	2245 (18.0%)
Resists Care	8564 (33.4%)	4597 (35.0%)	3967 (31.7%)
CHESS Scale Score, n (%)			
0	11,931 (46.5%)	5959 (45.3%)	5972 (47.8%)
1	8283 (32.3%)	4299 (32.7%)	3984 (31.9%)
2	3920 (15.3%)	2039 (15.5%)	1881 (15.0%)
3	1140 (4.4%)	621 (4.7%)	519 (4.2%)
4 or 5	379 (1.5%)	230 (1.8%)	149 (1.1%)
Diagnoses, n (%)			
Diabetes	7231 (28.2%)	3393 (25.8%)	3838 (30.7%)
Congestive heart failure	4346 (16.9%)	2045 (15.6%)	2301 (18.4%)
Alzheimer’s disease	4526 (17.6%)	2516 (19.1%)	2010 (16.1%)
Other dementia	10,873 (42.4%)	5793 (44.1%)	5080 (40.6%)
Stroke	5696 (22.2%)	2785 (21.2%)	2911 (23.3%)
Depression	7269 (28.3%)	3751 (28.5%)	3518 (28.1%)
Chronic Obstructive Pulmonary Disease	4870 (19.0%)	2305 (17.5%)	2565 (20.5%)
Cancer	2477 (9.7%)	1288 (9.8%)	1189 (9.5%)
Renal Failure	3117 (12.2%)	1388 (10.6%)	1729 (13.8%)
Number of chronic conditions, n (%)			
0 or 1	3292 (12.8%)	1725 (13.1%)	1567 (12.5%)
2	5601 (21.8%)	2934 (22.3%)	2667 (21.3%)
3	6346 (24.7%)	3289 (25.0%)	3057 (24.4%)
4	4965 (19.4%)	2524 (19.2%)	2441 (19.5%)
5+	5449 (21.2%)	2676 (20.4%)	2773 (22.2%)
Fell in prior 30 days, n (%)	4044 (15.8%)	2035 (15.5%)	2009 (16.1%)

### Emergency department visits

Index and repeat ED visits are described in Table 2. Of index ED visits, 25.3% were identified as potentially preventable, 20.0% as a fall-related injury, and 45.8% as other. At the end of the index ED visit, 56.5% of residents returned to their LTC home and 43.5% were admitted to hospital; those admitted to hospital had an average stay of 6.7 (8.4) days. Following the index visit, 19.1% died without subsequent ED use.

**Table 2:** Descriptive characteristics of index emergency department visit and subsequent repeat visits over one year

Visit Descriptors	Index ED visit	1st Repeat Visit	2nd Repeat Visit	3rd Repeat Visit	4th Repeat Visit	5th and subsequent repeat visits
	N = 25,653	N = 12,505	*N* = 5901	*N* = 2859	*N* = 1434	*N* = 1690
Type of ED visit, n (%)						
Potentially preventable	6479 (25.3%)	3380 (27.0%)	1697 (28.8%)	821 (28.7%)	422 (29.4%)	486 (28.8%)
Fall-related injury	5119 (20.0%)	1872 (15.0%)	723 (12.3%)	314 (11.0%)	138 (9.6%)	93 (5.5%)
Non-fall injury	829 (3.2%)	324 (2.6%)	125 (2.1%)	49 (1.7%)	18 (1.3%)	32 (1.9%)
Low Acuity	1458 (5.7%)	758 (6.1%)	297 (5.0%)	146 (5.1%)	79 (5.5%)	91 (5.4%)
Other	11,768 (45.9%)	6171 (49.3%)	3059 (51.8%)	1529 (53.5%)	777 (54.2%)	988 (58.5%)
Same ED Visit type as visit immediately preceding, n (%)	N/A	5452 (43.6%)	2556 (43.3%)	1497 (52.4%)	777 (54.2%)	1002 (59.3%)
Timing of ED visit relative to visit immediately preceding						
Immediate (< 3 days)	N/A	641 (5.1%)	371 (6.3%)	182 (6.4%)	102 (7.1%)	165 (9.8%)
Short-term (3-10 days)	N/A	1449 (11.6%)	830 (14.1%)	488 (17.1%)	253 (17.6%)	367 (21.7%)
Long-term (11-90 days)	N/A	5039 (40.3%)	2895 (49.1%)	1537 (53.8%)	816 (56.9%)	970 (57.4%)
Distant (> 90 days)	N/A	5376 (43.0%)	1805 (30.6%)	652 (22.8%)	263 (18.3%)	188 (11.1%)
ED Visit disposition, N (%)						
Admitted to hospital	11,163 (43.5%)	5657 (45.2%)	2762 (46.8%)	1318 (46.1%)	660 (46.0%)	709 (42.0%)
Died in ED	0 (0.0%)	100 (0.8%)	63 (1.1%)	22 (0.8%)	10 (0.7%)	12 (0.7%)
Returned to LTC	14,490 (56.5%)	6748 (54.0%)	3076 (52.1%)	1519 (53.1%)	764 (53.3%)	969 (57.3%)
Days in hospital among those admitted						
Mean ± SD	6.71 ± 8.44	7.18 ± 8.69	7.95 ± 14.16	7.27 ± 8.45	8.78 ± 19.07	8.16 ± 16.36
Median (IQR)	5 (3–8)	5 (3–9)	5 (3–9)	5 (3–9)	6 (3–10)	5 (2–9)
Died without making another ED Visit, n (%)	4899 (19.1%)	4139 (33.1%)	1957 (33.2%)	969 (33.9%)	464 (32.4%)	514 (30.4%)

Among first and second repeat ED visits, 43% were of the same type as the preceding visit, and concordance increased with subsequent ED visits. Of first repeat ED visits, 5.1% were immediate, 11.6% were short-term, 40.3% were long-term, and 43.0% were distant relative to the index ED visit. For later repeat ED visits, immediate, short-term, and long-term visit intervals became more frequent while distant intervals became less frequent. While the frequency of hospital admission changed little with successive repeat visits, the length of hospital stay among those admitted increased to an average of 8.2 (16.4) days and the proportion who died following the visit increased to over 30%. We looked at visit patterns stratified by index ED visit type but found little difference compared to the overall patterns (data not shown).

### Risk factors for repeat ED visits

Differences between residents who did and did not have at least one repeat ED visit were generally small (Table 1). Residents who made at least one repeat visit tended to be slightly younger, and have minimal cognitive impairment and ADL dependence than residents without repeat ED visits. There were also small differences across a number of diagnoses.

Results of both models to identify risk factors for repeat ED use are shown in Table 3. From the multinomial logistic regression, residents who were male, had moderate ADL dependence, any medical instability, diabetes, congestive heart failure, chronic obstructive pulmonary disease, renal failure, or dysrhythmia had a greater odds of making at least one repeat ED visit. Residents with longer lengths of stay, severe cognitive impairment, Alzheimer’s disease or other dementia, or 5 or more chronic conditions had a lower odds of making a repeat ED visit. There was no association with age; other variables, including fall history and specific diagnoses, were excluded from the final model. The complementary results on death from this multinomial logistic regression model are shown in Additional file 1: Table S1.

**Table 3:** Final logistic regression and Andersen-Gill regression model results to identify risk factors for any repeat ED visit and more frequent repeat ED visits, respectively

	Logistic regression	Andersen-Gill regression
	Odds ratio (95% Confidence interval)	Incidence rate Ratio (95% confidence interval)
Age		
65–74	REF	REF
75–84	1.01 (0.91–1.11)	1.03 (0.99–1.07)
85–94	1.00 (0.88–1.06)	1.05 (1.01–1.10)
95 and older	0.91 (0.79–1.04)	1.17 (1.10–1.25)
Sex		
Female	REF	REF
Male	1.27 (1.19–1.36)	1.20 (1.17–1.24)
Length of stay in LTC		
< 30 days	REF	REF
30–89 days	0.71 (0.56–0.89)	0.90 (0.83–0.97)
90–364 days	0.60 (0.49–0.73)	0.78 (0.73–0.83)
365 or more days	0.45 (0.38–0.57)	0.71 (0.67–0.76)
Cognitive impairment		
Minimal	REF	REF
Moderate	0.92 (0.86–1.01)	0.98 (0.95–1.01)
Severe	0.92 (0.84–0.99)	1.03 (0.99–1.07)
ADL dependence		
Minimal	REF	REF
Moderate	1.11 (1.02–1.21)	1.08 (1.04–1.13)
Dependent	1.07 (0.98–1.18)	1.23 (1.18–1.28)
CHESS scale score		
0	REF	REF
1	1.08 (1.01–1.15)	1.10 (1.06–1.13)
2	1.14 (1.05–1.24)	1.17 (1.13–1.21)
3	1.19 (1.02–1.39)	1.24 (1.16–1.31)
4 or 5	1.10 (0.83–1.44)	1.23 (1.11–1.37)
Diagnoses		
Diabetes	1.28 (1.19–1.37)	1.13 (1.10–1.16)
Congestive heart failure	1.26 (1.16–1.37)	1.14 (1.10–1.18)
Arthritis	0.93 (0.87–1.00)	0.93 (0.90–0.95)
Alzheimer’s disease or Other Dementia	0.93 (0.86–1.00)	0.95 (0.92–0.98)
Chronic obstructive pulmonary disease	1.26 (1.17–1.37)	1.11 (1.07–1.14)
Liver disease	–	1.09 (0.97–1.22)
Renal failure	1.35 (1.23–1.49)	1.16 (1.12–1.20)
Dysrhythmia	1.14 (1.03–1.27)	–
Cancer	1.06 (0.95–1.17)	–
Number of chronic conditions		
0 or 1	REF	REF
2	0.95 (0.85–1.05)	0.99 (0.95–1.04)
3	0.90 (0.81–1.01)	0.96 (0.92–1.01)
4	0.88 (0.78–1.00)	0.95 (0.90–1.00)
5 or more	0.82 (0.71–0.95)	0.94 (0.89–0.99)
Fell in prior 30 days	–	0.95 (0.92–0.98)

The A-G regression was used to model the rate of repeat ED visits. Preliminary modeling (not shown) did not indicate excess individual level heterogeneity in the number of repeat ED visits. Consistent with the results of the logistic regression, a higher rate of repeat ED visits was associated with being male, ADL dependence, medical instability, and specific diagnoses (diabetes, congestive heart failure, chronic obstructive pulmonary disease, and liver failure). Also consistent, residents with longer lengths of stay, Alzheimer’s disease or other dementia, and 5 or more chronic conditions had a lower relative rate of repeat ED visits. Unlike the logistic regression model, older age was associated with an increased rate of repeat ED visits while a prior fall was associated with a decreased rate. Neither cognitive impairment nor ADL dependence was associated with repeat ED visit rate.

### Frequent ED visitors

A total of 2859 (11.1%) residents were identified as frequent ED visitors (Table 4). Overall, frequent ED visitors tended to be younger, male, had shorter lengths of stay in the NH, to have minimal cognitive impairment and be independent in ADL function, and less likely to exhibit problem behaviours. Frequent ED visitors were more likely to have diabetes, congestive heart failure, chronic obstructive pulmonary disease, and renal failure but less likely to have Alzheimer’s disease or other dementias. In the final logistic regression model, similar patterns emerged. Notably, those who were older, had longer lengths of stay, or a diagnosis of Alzheimer’s disease or dementia had lower odds of being a frequent ED visitor, while residents who were male or had specific diagnoses (diabetes congestive heart failure, chronic obstructive pulmonary disease, renal failure, or liver disease) had greater odds of being a frequent ED user. No associations with cognitive impairment, ADL function, medical instability, number of chronic conditions, or behaviours were observed in the final model. We did find that residents whose index ED visit was for an injury (either fall or non-fall related) had reduced odds of being a frequent ED visitor. The results on death from this multinomial model are presented in Additional file 1: Table S2.

**Table 4:** Characteristics of frequent emergency department visitors (3 or more repeat ED visits) relative to non-frequent ED visitors and results of the logistic regression model to identify risk factors

	Non-Frequent ED visitors N – 22,794	Frequent ED visitors N = 2859	Adjusted Odds ratio (95% Confidence interval)
Age, n (%)			
65–74	2306 (10.1%)	476 (16.6%)	REF
75–84	7911 (34.7%)	1164 (40.7%)	0.90 (0.80–1.02)
85–94	10,696 (46.9%)	1088 (38.1%)	0.76 (0.67–0.87)
95 or older	1881 (8.3%)	131 (4.6%)	0.70 (0.57–0.88)
Sex, n (%)			
Female	15,703 (68.9%)	1801 (63.0%)	REF
Male	7091 (31.1%)	1058 (37.0%)	1.25 (1.14–1.37)
Length of stay in LTC, (%)			
< 30 days	649 (2.8%)	126 (4.4%)	REF
30–89 days	1031 (4.5%)	219 (7.7%)	0.96 (0.74–1.26)
90–364 days	5363 (23.5%)	851 (29.8%)	0.69 (0.55–0.86)
365 or more days	15,751 (69.1%)	1663 (58.2%)	0.51 (0.41–0.63)
Cognitive impairment, n (%)			
Minimal	10,246 (45.0%)	1605 (56.1%)	REF
Moderate	8140 (35.7%)	873 (30.5%)	0.92 (0.83–1.02)
Severe	4408 (19.3%)	381 (13.3%)	0.94 (0.81–1.10)
ADL Dependence, n (%)			
Minimal	3324 (14.6%)	518 (18.1%)	REF
Moderate	9504 (41.7%)	1281 (44.8%)	1.08 (0.96–1.22)
Dependent	9966 (43.7%)	1060 (37.1%)	1.05 (0.92–1.20)
Behaviours, n (%)			
Inappropriate behaviour	3933 (17.3%)	404 (14.1%)	0.90 (0.79–1.03)
Verbally abusive	4431 (19.4%)	523 (18.3%)	1.11 (0.98–1.26)
Physically abusive	2618 (11.5%)	241 (8.4%)	0.92 (0.77–1.09)
Wandering	4216 (18.5%)	425 (14.9%)	0.91 (0.80–1.04)
Resists care	7734 (33.9%)	830 (29.0%)	0.99 (0.89–1.10)
CHESS Scale Score, n (%)			
0	10,552 (46.3%)	1379 (48.2%)	REF
1	7400 (32.5%)	883 (30.9%)	1.03 (0.94–1.13)
2	3470 (15.2%)	450 (15.7%)	1.20 (1.06–1.35)
3	1031 (4.5%)	109 (3.8%)	1.18 (0.95–1.48)
4 or 5	341 (1.5%)	38 (1.3%)	1.27 (0.87–1.85)
Diagnoses, n (%)			
Diabetes	6220 (27.3%)	1011 (35.4%)	1.28 (1.16–1.42)
Congestive Heart Failure	3725 (16.3%)	621 (21.7%)	1.40 (1.24–1.57)
Arthritis	9640 (42.3%)	1146 (40.1%)	0.90 (0.82–1.00)
Osteoporosis	6554 (28.8%)	714 (25.0%)	0.92 (0.83–1.03)
Alzheimer’s disease	4159 (18.3%)	367 (12.8%)	0.83 (0.74–0.92)
Other dementias	9845 (43.2%)	1028 (36.0%)	-*
Stroke	5026 (22.0%)	670 (23.4%)	–
Anxiety disorder		254 (8.9%)	1.00 (0.86–1.16)
Depression	6474 (28.4%)	795 (27.8%)	0.95 (0.85–1.05)
Chronic obstructive pulmonary disease	4201 (18.4%)	669 (23.4%)	1.25 (1.12–1.40)
Cancer	2216 (9.7%)	261 (9.1%)	1.06 (0.91–1.23)
Renal Failure	2644 (11.6%)	473 (16.5%)	1.38 (1.22–1.57)
Liver disease			1.35 (0.95–1.92)
Number of chronic conditions, n (%)			
0 or 1	2919 (12.8%)	373 (13.0%)	REF
2	5000 (21.9%)	601 (21.0%)	0.95 (0.81–1.11)
3	5675 (24.9%)	671 (23.5%)	0.92 (0.78–1.09)
4	4455 (19.5%)	510 (17.8%)	0.82 (0.68–1.00)
5 or more	4745 (20.8%)	704 (24.6%)	0.92 (0.73–1.16)
Index Visit Type, n (%)			
Potentially preventable			REF
Fall-related injury			0.66 (0.57–0.75)
Non-fall injury			0.75 (0.59–0.97)
Low acuity			0.95 (0.80–1.14)
Other			0.97 (0.87–1.07)

*a single variable for Alzheimer's disease or other dementias was used in the logistic regression model

## Discussion

In our comprehensive, population-based cohort study, we found that repeated transfers to the ED were common among NH residents. Within one year, nearly half of residents returned to the ED at least once, and 11% returned 3 or more times. We found that 40–60% of return visits were for reasons similar to the preceding visit and that the majority occurred at least 10 days after the prior visit. At the end of each visit, more than half of residents were discharged back to their NH and the proportion who died following a visit nearly doubled over time.

Our data show that there were generally small differences between residents who did and did not experience repeat ED visits. Overall, these groups were fairly similar with only slight differences in age, sex, chronic conditions, fall history, and behaviours, with more noticeable differences on specific diagnoses and cognitive and physical impairment. Following extensive modeling, certain characteristics emerged as risk factors but no single variable (or group of variables) were strongly predictive. Notably, those who were male and were clinically unstable, as measured by the CHESS scale, had a higher likelihood of repeat ED visits. These findings suggest what might be expected – that those residents with greater, and potentially more complex, medical need are more likely to have frequent encounters with acute care. Taken with the increased frequency of death following a greater number of ED visits, it is also possible that residents were more likely to experience repeat transfers as they approached end-of-life. In context with the rest of our findings, though, this appears to be only part of the story.

We also found that 11% of residents met the criteria for being a frequent ED visitor (3 or more repeat ED visits). Similar to what has been observed in the non-LTC population, we found that a small group (estimated at 4% of all residents aged 65 or older) accounted for a large share of repeat ED use. These residents were more likely to be younger than 75, male, have relatively shorter lengths of stay, and have minimal cognitive impairment. They were also more likely to be diagnosed with congestive heart failure, chronic obstructive pulmonary disease, diabetes, and renal failure but less likely to have Alzheimer’s disease or other dementias. Many of these same variables, notably length of stay and the diagnoses, were also associated with a greater rate of repeat ED visits. This group of frequent ED visitors is very different from the general picture of LTC residents who are most frequently characterized as very old, female, and cognitively impaired due to dementia [20, 21]. To date, there is little data to describe this subgroup and how they differ from the larger population of residents or why they are so frequently transferred to the ED.

We used two approaches to identify risk factors for repeat ED visits. While these two approaches generally revealed consistent results, others were quite different. This is not unexpected given that one approach identified risk factors associated with the likelihood of at least one repeat ED visit (logistic regression) while the other identified risk factors associated with the rate of repeat ED visits (Andersen-Gill regression). These divergent findings also may reflect the heterogeneity among NH residents at risk for experiencing repeat ED visits – and, potentially, different patterns of repeat ED visits. Our data suggests at least two groups of residents at risk for repeat ED visits – those who are medically complex (possibly approaching the end of life) and those who are younger without cognitive impairment. A better understanding of these subgroups and their needs is critical to the successful implementation of interventions to safely reduce ED transfers and improve NH care.

This study has a number of limitations. First, we included only residents over age 65 since these residents tend to differ from others [22]. We found, however, that frequent ED use was most common among residents 65–74 years, an age group typically considered “young” in NH settings. This raises questions about the needs of the 65–74 age group and whether they may be more reflective of even younger residents, who may age within NHs, than older residents who tend to enter at later stages of illness. Second, despite the breadth of data, we had very little information on the factors immediately preceding an ED transfer, such as acute changes, family and resident preferences, or how a prior transfer influences subsequent transfer. Our study offers a broad overview of the problem of repeat ED transfers but additional research is required to explore the immediate details. Third, we used data from a single jurisdiction and it is difficult to know how generalizable our findings are to other jurisdictions given how little available data there is on repeat ED visits. Given that NH populations are fairly similar across jurisdictions and ED transfers are common, we anticipate that our overall trends are generalizable but the individual risk factors may differ. Finally, this research focuses solely on resident characteristics and does not include information on either the NH or the treating ED. Given that this is among the first studies to explore repeat ED transfers, we intentionally focused on residents; however, it is clear from other research that facility characteristics do influence resident outcomes, including acute care use [16, 23].

## Conclusions

In this study of over 25,000 NH residents, we found nearly half made at least one repeat ED and 11% made at least three repeat ED visits. There were subtle differences between residents who did and did not experience repeat visits but our findings suggest that medically complex residents and younger residents without cognitive impairment are at risk for such outcomes. Reducing ED use, especially repeated use, will require a better understanding of these resident groups and how to best meet their needs within the NH environment.

## Additional file

Additional file 1: Multinomial Model Results for DeathG (DOCX 25 kb)

## Abbreviations

ADL: Activities of Daily Living
A-G model: Andersen-Gill regression model
CCRS: Continuing Care Reporting System
CHESS: Changes in Health and End-Stage Signs and Symptoms
DAD: Discharge Abstract Database
ED: Emergency department
ICES: Institute for Clinical Evaluative Sciences
NACRS: National Ambulatory Care Reporting System
NH: Nursing home
RAI-MDS 2.: Resident Assessment Instrument Minimum Data Set version 2.0
RPDB: Registered Persons Database
