# Supplementary material for: Repeat emergency department visits by nursing home residents: a cohort study using health administrative data
Source: BMC Geriatr. 2018 Jul 5;18:157. doi: 10.1186/s12877-018-0854-8 (PMC6034297; doi:10.1186/s12877-018-0854-8)
Supplement: Supplementary file 1 — Multinomial Model Results for DeathG (DOCX 25 kb) [file 12877_2018_854_MOESM1_ESM.docx]

Additional file 1: Table S1: Odds of Death (without a Repeat Emergency Department Visit) relative to No Repeat Emergency Department Visit During the One Year Following the Index Emergency Department Visit*

| Risk Factor | Odds Ratio | 95% Confidence Interval |
| --- | --- | --- |
| Age |  |  |
| 65-74 | REF | REF |
| 75-84 | 1.52 | 1.32-1.76 |
| 85-94 | 2.07 | 1.79-2.38 |
| 95 and older | 3.27 | 2.74-3.90 |
| Sex |  |  |
| Female | REF | REF |
| Male | 1.54 | 1.42-1.67 |
| Length of stay in LTC |  |  |
| <30 days | REF | REF |
| 30-89 days | 0.77 | 0.58-1.02 |
| 90-364 days | 0.59 | 0.47-0.75 |
| 365 or more days | 0.58 | 0.46-0.72 |
| Cognitive Impairment |  |  |
| Minimal | REF | REF |
| Moderate | 1.10 | 1.00-1.21 |
| Severe | 1.33 | 1.19-1.50 |
| ADL Dependence |  |  |
| Minimal | REF | REF |
| Moderate | 1.37 | 1.21-1.56 |
| Dependent | 2.29 | 2.01-2.61 |
| CHESS Scale Score |  |  |
| 0 | REF | REF |
| 1 | 1.46 | 1.34-1.59 |
| 2 | 1.72 | 1.54-1.90 |
| 3 | 2.12 | 1.79-2.52 |
| 4 or 5 | 2.79 | 2.11-3.69 |
| Diagnoses |  |  |
| Diabetes | 1.29 | 1.84-1.42 |
| Congestive Heart Failure | 1.22 | 1.16-1.44 |
| Arthritis | 0.90 | 0.82-0.98 |
| Alzheimer’s disease or Other Dementia | 0.99 | 0.90-1.09 |
| Chronic Obstructive Pulmonary Disease | 1.37 | 1.24-1.52 |
| Renal Failure | 1.23 | 1.08-1.38 |
| Dysrhythmia | 1.22 | 1.07-1.40 |
| Cancer | 1.39 | 1.23-1.57 |
| Number of Chronic Conditions |  |  |
| 0 or 1 | REF | REF |
| 2 | 0.95 | 0.83-1.09 |
| 3 | 0.77 | 0.67-0.89 |
| 4 | 0.77 | 0.66-0.91 |
| 5 or more | 0.64 | 0.53-0.77 |

NOTE: 4,897 residents died without making a repeat emergency department visit; 8,249 were in the reference category (neither died nor made a repeat visit).

*These results are complementary to those found in Table 3 of the main text.

Additional file 1: Table S2: Odds of Death Relative to Not Having Frequent Emergency Department Visits in the One-Year following the Index Emergency Department Visit*

|  | Odds Ratio | 95% Confidence Interval |
| --- | --- | --- |
| Age |  |  |
| 65-74 | REF | REF |
| 75-84 | 1.57 | 1.41-1.74 |
| 85-94 | 2.20 | 1.98-2.44 |
| 95 or older | 3.58 | 3.12-4.10 |
| Sex |  |  |
| Female | REF | REF |
| Male | 1.42 | 1.34-1.52 |
| Length of stay in LT |  |  |
| <30 days | REF | REF |
| 30-89 days | 0.81 | 0.66-0.99 |
| 90-364 days | 0.66 | 0.55-0.78 |
| 365 or more days | 0.72 | 0.61-0.84 |
| Cognitive impairment |  |  |
| Minimal | REF | REF |
| Moderate | 1.15 | 1.07-1.24 |
| Severe | 1.41 | 1.29-1.55 |
| ADL Dependence |  |  |
| Minimal | REF | REF |
| Moderate | 1.39 | 1.27-1.52 |
| Dependent | 2.06 | 1.87-2.27 |
| Behaviours |  |  |
| Inappropriate behaviour | 1.02 | 0.93-1.10 |
| Verbally abusive | 1.00 | 0.91-1.08 |
| Physically abusive | 1.06 | 0.95-1.18 |
| Wandering | 0.89 | 0.820.97 |
| Resists care | 1.04 | 0.97-1.11 |
| CHESS Scale Score |  |  |
| 0 | REF | REF |
| 1 | 1.28 | 1.20-1.37 |
| 2 | 1.57 | 1.44-1.70 |
| 3 | 2.06 | 1.79-2.36 |
| 4 or 5 | 2.15 | 1.70-2.72 |
| Diagnose |  |  |
| Diabetes | 1.12 | 1.04-1.20 |
| Congestive Heart Failure | 1.26 | 1.16-1.37 |
| Arthritis | 0.89 | 0.84-0.95 |
| Osteoporosis | 0.94 | 0.87-1.00 |
| Alzheimer’s disease or other Dementias | 0.99 | 0.92-1.07 |
| Anxiety disorder | 0.87 | 0.78-0.97 |
| Depression | 0.93 | 0.86-0.99 |
| Chronic obstructive pulmonary disease | 1.19 | 1.10-1.28 |
| Cancer | 1.52 | 1.38-1.68 |
| Renal Failure | 1.21 | 1.10-1.32 |
| Liver disease | 0.88 | 0.65-1.18 |
| Number of chronic conditions |  |  |
| 0 or 1 | REF | REF |
| 2 | 1.00 | 0.90-1.11 |
| 3 | 0.92 | 0.82-1.03 |
| 4 | 0.94 | 0.82-1.07 |
| 5 or more | 0.84 | 0.72-0.99 |
| Index Visit Type |  |  |
| Potentially preventable | REF | REF |
| Fall-related injury | 0.53 | 0.48-0.58 |
| Non-fall injury | 0.46 | 0.38-0.54 |
| Low acuity | 0.53 | 0.47-0.61 |
| Other | 0.90 | 0.84-0.96 |

NOTE: 9,605 residents died without meeting the criteria for making frequent emergency department visits (3 or more repeat visits within the follow-up year); 13,187 residents were in the reference category (neither died nor met the criteria for making frequent emergency department visits).

*These results are complementary to those presented in Table 4.
